# Study of the Structure, Electronic and Optical Properties of BiOI/Rutile-TiO_2_ Heterojunction by the First-Principle Calculation

**DOI:** 10.3390/ma13020323

**Published:** 2020-01-10

**Authors:** Zhan Qu, Yali Su, Li Sun, Feng Liang, Guohe Zhang

**Affiliations:** 1 School of Microelectronics, Xi’an Jiaotong University, Xi’an 710049, China; zhanqu@snnu.edu.cn (Z.Q.); sl-lxa@mail.nwpu.edu.cn (L.S.); 2 School of Mechanical Engineering, Xi’an Shiyou University, Xi’an 710065, China; sylemon@163.com

**Keywords:** heterojunction, electronic structure, band offset, photoresponse, first-principles

## Abstract

Using the first-principle calculation that is based on the density functional theory (DFT), our group gains some insights of the structural, electronic and optical properties of two brand new types of BiOI/TiO_2_ heterojunctions: 1I-terminated BiOI {001} surface/TiO_2_ (1I-BiOI/TiO_2_) and BiO-terminated BiOI {001} surface/TiO_2_ (BiO-BiOI/TiO_2_). The calculation illustrates that BiOI/TiO_2_ heterojunction has excellent mechanical stability, and it shows that there is a great possibility for the BiOI/TiO_2_ heterojunction to be used in visible-light range, hence the photocatalytic ability can be enhanced dramatically. Especially, from the calculation, we discovered that there are two specific properties: the band-gap of 1I-BiOI/TiO_2_ heterojunction reduces to 0.28 eV, and the BiO-BiOI/TiO_2_ semiconductor material changes to n-type. The calculated band offset (BOs) for 1I-BiOI/TiO_2_ heterojunction indicates that the interfacial structure contributes a lot to a suitable band alignment which can disperse the photo-generated carriers into the opposite sides of the interface, so this could effectively weaken the electron-hole recombination. Meanwhile, the built-in potential around the interface accelerates the movement of the photo-generated electron-hole pairs. We believe this is the reason that the BiOI/TiO_2_ material shows perfect photocatalytic performance. This paper can provide theoretical support for the related research, especially the further research of the BiOI-based material.

## 1. Introduction

In modern environmental engineering, photocatalyst has been widely used for activities such as pollution degradation. Titania (TiO_2_) is a typical candidate, and it has received much attention. Besides environmental engineering, it has been used in many fields like photovoltaics. TiO_2_ has perfect properties, such as non-toxic, low-cost and great chemical stability [1,2,3]. But for the energy band properties, TiO_2_ is a wide band-gap oxide-semiconductor (3.0 eV for rutile, 3.2 eV for anatase), shows photocatalytic activity under the ultra-violet part of the solar spectrum (λ < 384 nm). This accounts for only a small portion (5%) of the solar energy. This is the reason that the wide bandgap greatly limits its performance of photocatalytic-degradation [4]. Besides, the rapid recombination of photo-induced electron-hole pairs greatly reduces the quantum efficiency of the system and making its photo-generated quantum-yield to be very low [5,6,7,8,9]. Therefore, it is essential for the researchers to develop effective methods of narrowing the band-gap to the visible-light range and improving the charge-separation efficiency of TiO_2_. Therefore, it is highly possible that TiO_2_ could be used as an excellent photocatalyst for environmental engineering.

Forming two different semiconductor layers into a heterostructure is an effective method to improve its properties. Under suitable energy band arrangement, the interface could transfer the carriers from a lower energy band to a higher energy band. Meanwhile, the heterojunction helps the separation of photo-induced electron-hole pairs and prevents the recombination of electron-hole pairs, which could greatly enhance the photocatalytic efficiency of semiconductor heterostructure. In previous researches, scientists found that constructing an interface structure of TiO_2_ with a narrow-gap material, such as CdS [10,11], CdSe [12,13], CdTe [10], and Cu_2_O [14,15] was considered as a possible method. Under visible-light irradiation, the electron excited from the narrower bandgap semiconductor then transfers to the conduction band of TiO_2_, which can intensify the carrier’s transfer and enhances the photocatalytic ability significantly [15,16,17].

Zhang et al. applied a new approach to compose the bismuth oxyhalide BiOX (X = Cl, Br, I) nanospheres [18]. The BiOX with a special layer structure exhibits excellent catalytic properties of degrading pollutants under visible light irradiation. Among those BiOX structures, BiOI is a kind of typical p-type semiconductor with a narrow bandgap of 1.85 eV. It has drawn a lot of attention due to its excellent mechanical and catalytic properties [19,20,21,22]. However, its application is affected by the high carrier-recombination probability. This is a severe bottleneck for the use of BiOI. Due to all these reasons, we hypothesize that the combination of BiOI and TiO_2_ into a heterojunction might be a wonderful method to overcome the different disadvantages of the two kinds of semiconductors.

In previous experiments, BiOI/TiO_2_ heterojunction has been synthesized and demonstrated excellent photocatalytic performance. It is shown that the performance is better than single BiOI or single TiO_2_ [4,23]. However, there is limited theoretical research on BiOI/TiO_2_ heterojunction. Especially on the aspect of structural stability, electronic structure, and photocatalytic properties. Due to the absence of relevant theoretical modeling research, it is difficult to further the research of the structure and the photo-catalytic ability. Therefore, we make a theoretical study based on the first-principle method to explore the photocatalytic mechanism of BiOI/TiO_2_ heterojunctions in this paper.

## 2. Computation Detail and Models

This research is conducted using Materials Studio software (CASTEP) based on the DFT method. Perdew-Burke-Emerhof PBE function of the generalized gradient approximation (GGA) method is used to process the exchange correlation energy [24,25]. A plane-wave cutoff energy (Ecutoff) of 410 eV and ultra-soft pseudo-potential are adopted in the reciprocal K space. A 6 × 6 × 1 Monkhorst–Pack [26] mesh grid is found to be enough to reach convergence for bulk supercell calculations. All the atoms of the heterojunction are fully relaxed to their equilibrium positions with an energy convergence of 5 × 10^−6^ eV and the atomic displacement is less than 5 × 10^−4^ Å. The calculated parameters have been tested to be credible to get a convergent result in the work. The pseudo-potentials used for the heterojunction are constructed by the electron configurations as Bi-6s6p, O-2s2p, I-5s5p, and Ti-3d4s for the ground-state of electronic structure calculations [27].

**Figure 1:**
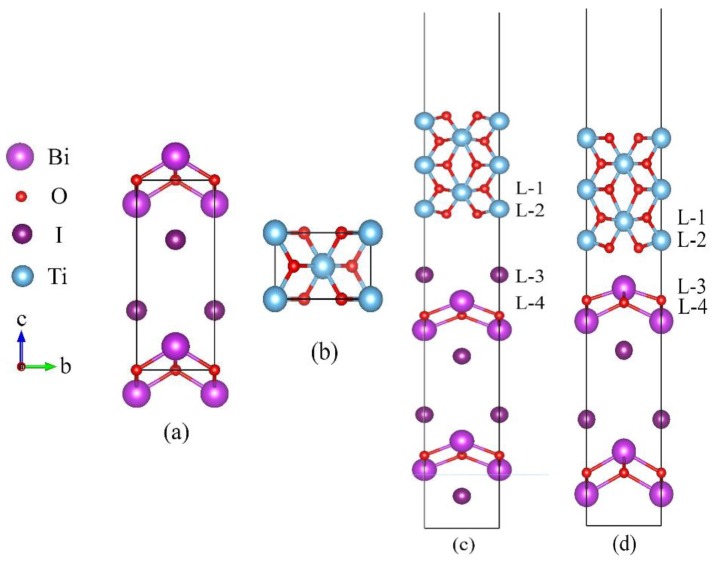
Crystal models: (**a**) BiOI, (**b**) rutile-TiO_2_, finally relaxed configuration of (**c**) 1I-BiOI/TiO_2_ and (**d**) BiO-BiOI/TiO_2_ heterojunction. Purple, red, brown and blue spheres represent Bi, O, I and Ti atoms respectively. L-1 to L-4 represent atomic layer from layer-1 to layer-4.

TiO_2_ exists in three crystalline polymorphs (anatase, rutile, and brookite). Figure 1a,b shows the structures of BiOI and rutile-TiO_2_ (TiO_2_) respectively. For TiO_2_, the lattice constants are a = b = 4.594 Å, c = 2.960 Å, α = β = γ = 90° [28]. For BiOI, the lattice constants of BiOI are a = b = 3.984 Å, c = 9.128, α = β = γ = 90° [29,30]. Notably, the BiOI is constituted of [Bi_2_O_2_]^2+^ and 2I^2−^ layers through weak Van der Waals forces. So, the BiOI unit cell is prone to be dissociated along the {001} direction. In a recent study, Kong et al. [31] studied the properties of three different sections for BiOI {001} surface ({001}-1I, {001}-BiO and {001}-2I) by using first-principles calculations based on DFT within GGA scheme. The calculations results indicate that the {001}-1I facet exhibits the smallest surface energy and the best structural stability. Meanwhile, {001}-BiO facet shows the best photocatalytic performance. Those aspects indicate that 1I-BiOI {001} and BiO-BiOI {001} surfaces are the noteworthy model to compose of a heterojunction with TiO_2_. For this reason, 1I-terminated and BiO-terminated of BiOI {001} surface is attempted to construct heterojunction with {001} crystal plane of rutile-TiO_2_.

It demonstrates that the geometry structures of BiOI {001} surface and TiO_2_ {001} surface are equally square. To get a higher matching of BiOI {001} and TiO_2_ {001} surfaces, the lattice parameters of the new heterostructure are chosen to be a = b = 4.289 Å, which is the average value of the two separate lattice parameters. Thus, the mismatch is only 6.63% for BiOI {001} crystal (along with the X and Y directions). The lattice parameters of the heterojunctions are fixed, whereas the atoms in the materials can relax fully optimization in the calculation. 1I-terminated BiOI {001} surface/TiO_2_ (1I-BiOI/TiO_2_) and BiO-terminated BiOI {001} surface/TiO_2_ (BiO-BiOI/TiO_2_) heterojunctions are shown in Figure 1c,d. A 15 Å vacuum region is set to avoid interactions between the top and bottom atoms in the periodic slab images of 1I-BiOI/TiO_2_ and BiO-BiOI/TiO_2_ heterojunctions. For 1I-BiOI/TiO_2_ heterojunction, the whole slab is 38.39 Å contains 25 atoms. The BiOI slab is composed of four layers of Bi atoms, two layers of O atoms and two layers of I atoms. The TiO_2_ slab is composed of five atomic layers contain five layers of Ti atoms and five layers of O atoms. For BiO-BiOI/TiO_2_ heterojunction, the whole slab is 44.56 Å containing 27 atoms. The BiOI slab is composed of four layers of Bi atoms, two layers of O atoms and four layers of I atoms, and the TiO_2_ slab has five atomic layers consisting of five layers of Ti atoms and five layers of O atoms. Finally, the structures, electronic and optical properties of 1I-BiOI/TiO_2_ and BiO-BiOI/TiO_2_ heterojunctions are systematically investigated according to the density functional theory.

## 3. Results and Discussion

### 3.1. The Geometric Structures and Formation Energy of BiOI/TiO_2_ Heterojunction

The theoretically optimized model of both 1I-BiOI/TiO_2_ and BiO-BiOI/TiO_2_ heterojunction is shown in Figure 1c,d.

For the 1I-BiOI/TiO_2_ interface (Shown in Figure 1c), the Ti atoms in L-2 are bonded with I atoms in L-3, and the bond length is 4.80 Å. The O atoms in layer-2 are bonded with I atoms in L-3, and the O-I bond length is 4.23 Å. Comparing with unrelaxed heterojunction, the bond length of Ti-I increases by 0.74 Å, and the bond length of O-I increases by 0.75 Å. Meanwhile, the O (L-1)-Ti (L-2) bond length decreases by 0.09 Å, and I (L-3)-Bi (L-4) bond length also decreases by 0.09 Å. The Bi-O bond length and Ti-O bond length away from BiOI/TiO_2_ interface increase slightly.

For the BiO-BiOI/TiO_2_ heterojunction, as shown in Figure 1d, Ti atoms in L-2 are bonded with O atoms in L-4, and the Ti-O bond length of 3.34 Å. O atoms in L-2 are bonded with Bi atoms in L-4, and the bond length is 2.70 Å. Comparing to the unrelaxed BiO-BiOI/TiO_2_ heterojunction, the bond length of Ti-O decreases by 0.23 Å, and the bond length of Bi-O decreases by 0.13 Å. Meanwhile, O (L-1)-Ti (L-2) bond length decreases slightly by 0.09 Å, while Bi (L-3)-O (L-4) bond length also increases by 0.14 Å. Similarly, the Bi-O and Ti-O bond length away from BiOI/TiO_2_ interface also increases slightly. When the two materials are combined, the atoms on the BiOI/TiO_2_ interface of different materials can generate a force that includes repulsion and attraction, and this will change the bond length of atoms. After the optimization, the bond lengths of Bi-O and Ti-O all increase slightly in the BiOI/TiO_2_ heterojunction.

For the BiO-BiOI/TiO_2_ heterojunction, as shown in Table 1, the Mulliken’s bonding population of O (L-2)-Bi (L-4) bond is 0.08 eV, indicating that Bi-O bonds at the interface have a large part of ionic properties and little covalent properties. Another bond population of Bi-O and Ti-O bonds in the BiOI/TiO_2_ interface is zero, which means that ionic property dominates in those bonds.

To contrast the structural stability of those two BiOI/TiO_2_ heterojunctions, we calculate the formation energy ($E_{\mathrm{form}}$) using the following formula [32]:

(1) $$E_{\mathrm{form}}=\frac{E_{\mathrm{total}}-(E_{BiOI}{+n}_{Ti}\mu_{Ti}{+n}_{O}\mu_{O})}{A}$$

$E_{\mathrm{total}}$ and $E_{BiOI}$ represent the calculated DFT energy for the 1I or BiO terminated BiOI {001} surface model; $n_{Ti}$ and $n_{O}$ represent the numbers of Ti and O atom in TiO_2_ slab; $\mu_{Ti}$ and $\mu_{O}$ are the chemical-potential of the Ti and O atoms; A represents the cross-section area of the interface for the BiOI/TiO_2_ heterojunction; $\mu_{Ti}$ represents the Ti atomic energy in the bulk Ti; $\mu_{O}$ represents half of the total energy of oxygen molecules. The calculated formation energy $E_{\mathrm{form}}$ of 1I/TiO_2_ and BiO/TiO_2_ heterojunctions are −2.21 eV/Å^2^ and −2.25 eV/Å^2^, respectively. The $E_{\mathrm{form}}$ of the heterojunctions are negative indicating the formations of BiOI/TiO_2_ heterojunctions are exothermic and spontaneous, and BiOI and TiO_2_ can easily combine. Furthermore, it can be found that BiO-BiOI/TiO_2_ is more stable than the 1I-BiOI/TiO_2_ heterojunction.

### 3.2. Electronic Properties

#### 3.2.1. Electronic Structure of BiOI/TiO_2_ Heterojunction

**Figure 2:**
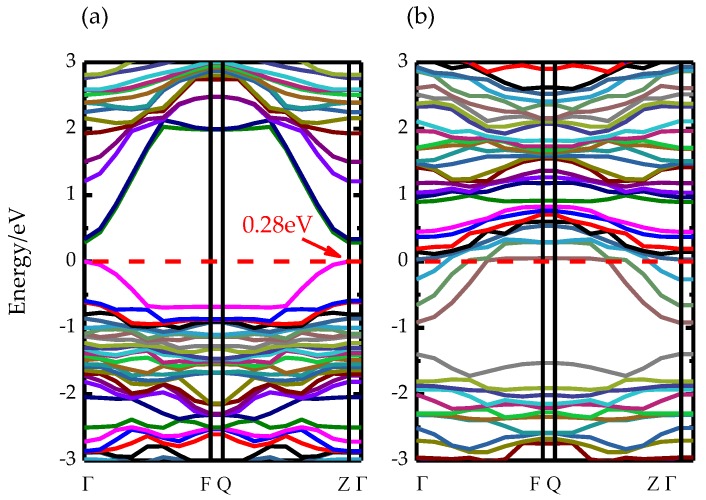
The band structures of relaxed (**a**) 1I-BiOI/TiO_2_, (**b**) BiO-BiOI/TiO_2_ heterojunction. Red dashed line represents the Fermi level.

The band structures are computed along the special lines connecting the following high-symmetry points: Γ(0,0,0), F(0,0.5,0), Q(0,0.5,0.5), Z(0,0,0.5) and Γ(0,0,0). Figure 2 demonstrates the band structures for the two heterojunctions. It shows that the conduction band maximum (CBM) for the two types of heterojunctions is at Γ point, while the conduction band minimum (VBM) is at Z point. This indicates the typical characteristic of indirect bandgap material [31]. The band-gap of 1I-BiOI/TiO_2_ heterojunction is 0.28 eV. This band-gap is decreased a lot compared to the pure BiOI or the TiO_2_. While for BiO-BiOI/TiO_2_ heterojunction, the Fermi level is located at the CBM which means it exhibits the property of n-type semiconductor. A smaller band-gap can produce the redshift phenomenon for 1I-BiOI/TiO_2_ heterojunction.

Due to the lattice mismatch, there exist two kinds of strains. The in-plane biaxial tensile strain (ε > 0) and compressive strain (ε < 0). The strain in the {001} plane of the interface between the BiOI and TiO_2_ is induced by the relative difference of lattice constants. The in-plane biaxial tensile strains (IPBTSs) is calculated by the following formula:

(2) $$\varepsilon =\frac{a-a_{0}}{a_{0}}=\frac{\Delta a}{a_{0}}$$

where a and $a_{0}$ denote the modified and unmodified lattice constants of the 1I-BiOI/TiO_2_ heterojunction respectively. To understand the influence of strain on the electronic properties of 1I-BiOI/TiO_2_ heterojunction, we applied a {001} IPBTSs to it. The applied strains vary from −2% to 2% by changing the lattice parameters of 1I-BiOI/TiO_2_ heterojunction.

**Figure 3:**
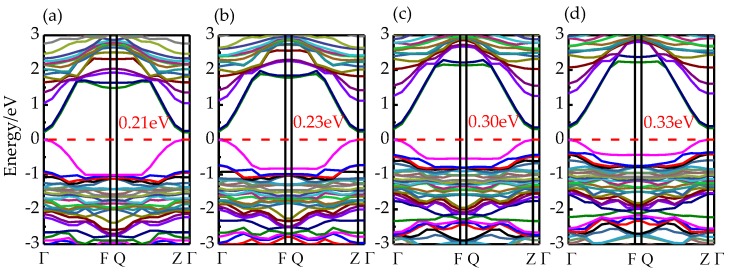
The band structures of 1I-BiOI/TiO_2_ heterojunction under (**a**) −2%, (**b**) −1%, (**c**) 1% and (**d**) 2% {001} IPBTSs. Red dashed line represents the Fermi level.

Figure 3 demonstrates the band structures of the 1I-BiOI/TiO_2_ heterojunction under different {001} IPBTSs. The band gaps are 0.21 eV, 0.23 eV, 0.30 eV and 0.33 eV for −2%, −1%, 1% and 2% IPBTSs respectively. Notably, the band gaps of those modified materials increase with the increasing strain of the 1I-BiOI/TiO_2_ heterojunction; meanwhile, the variation of the band-gap changes are all in the range of 0.12 eV. Therefore, the result of the band gap in our calculation is reliable.

**Figure 4:**
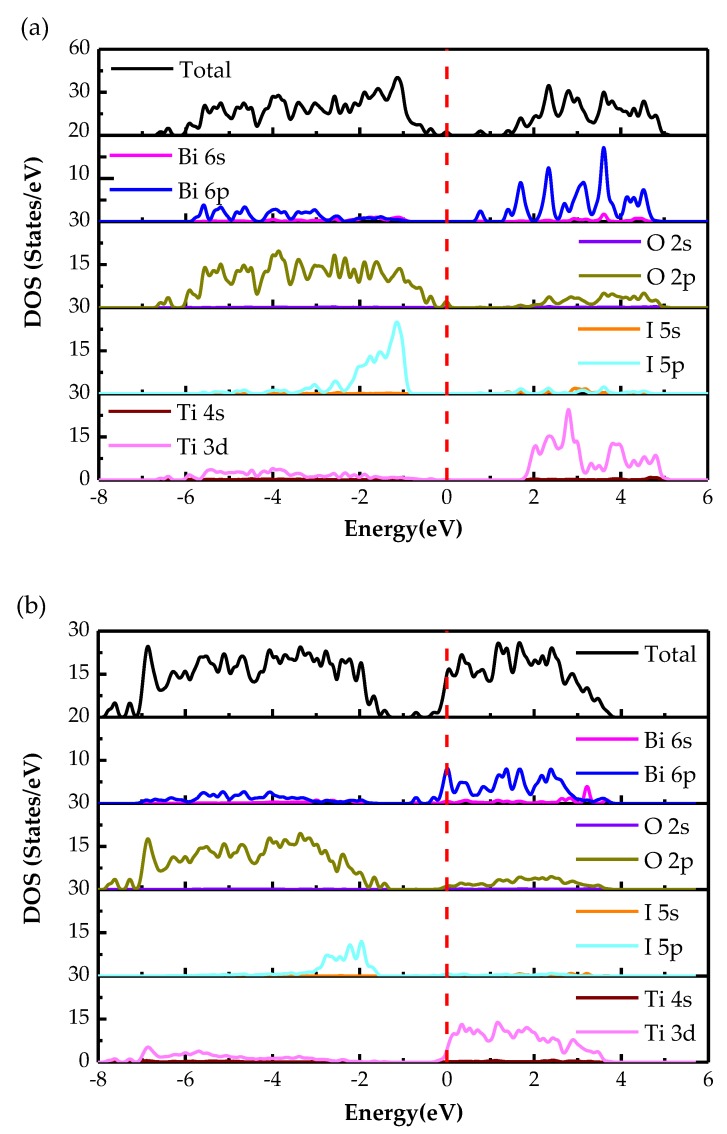
The total and partial density of states of (**a**) 1I-BiOI/TiO_2_, (**b**) BiO-BiOI/TiO_2_ heterojunctions. Red dashed line represents the Fermi level.

The total and partial density of states (DOS) for the two heterojunctions depict in Figure 4. It shows the difference between TDOS and PDOS for those heterojunctions. As we can see, in Figure 4, for those two types of heterojunctions, VB is primarily made up of I 5p, O 2p and little Bi 6p states and the VBM is primarily made up of O 2p states, while the CB is chiefly consisted of Bi 6p, Ti 3d, O 2p states. The CBM primarily consists of Bi 6p and Ti 3d states. It reveals that the reason why the photo response for the 1I-BiOI/TiO_2_ heterojunction is that the electron transfers from the O 2p in VB to the hybridized Bi 6p and Ti3d states in the CB. Figure 4b presents the DFT-calculated TDOS and PDOS of BiO-BiOI/TiO_2_ heterojunction. It can be seen that the CBM downward movement 1.10 eV contrasts to the 1I-BiOI/TiO_2_ heterojunction promoting the Fermi level through the CB and makes it an n-type semiconductor.

#### 3.2.2. Band Offset and Charge Transfer

When two kinds of semiconductors combine into a heterojunction, the band structure of the interface in the heterojunction will form a discontinuous condition. The discontinuous condition finally forms band offset (BOs) (i.e., valence band offset (VBO) and conduction band offset (CBO)). BOs is the most important factor to influence the photo-generated carriers’ movement through the interface. Two methods can be applied to obtain the BOs; first is the “local density of states (LDOS)” [33,34] and second is the “potential-line-up methods” [28,35,36]. Here, we use the first approach to obtain the Bos. The second approach is also applied to verify the precision of the BOs of the 1I-BiOI/TiO_2_ heterojunction.

**Figure 5:**
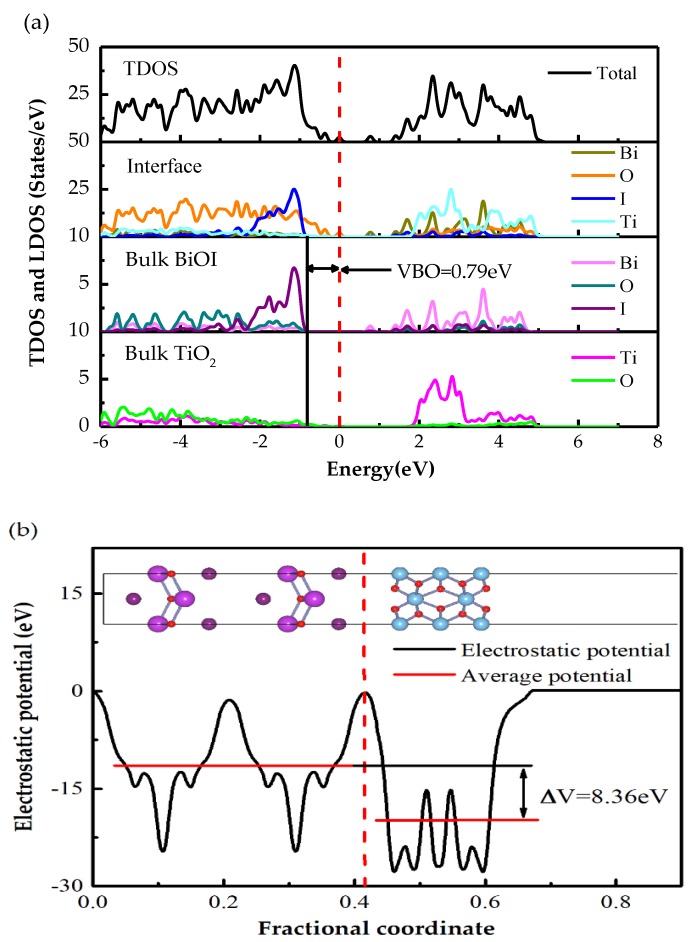
(**a**) The total and local density of states, (**b**) electrostatic potential of the 1I-BiOI/TiO_2_ heterojunction.

The DFT method calculated the density of states, including the TDOS and LDOS regarding bilateral layers far from the interface of the 1I-BiOI/TiO_2_ heterojunction that is plotted in Figure 5a. The bulk BiOI and bulk TiO_2_, shown in Figure 5a represents the layers far from the interface indicating that the electronic distribution is like that in independent materials. It can be seen from Figure 5a that the valence band and conduction band of bulk BiOI shows a larger shift to the lower energy. In addition, it can be perceived that the band-gap of bulk BiOI is about 1.39 eV, ranging from −0.79 to 0.60 eV, while bulk TiO_2_ has a band-gap of 1.91 eV, ranging from 0 to 1.91 eV. The band gap of bulk BiOI and TiO_2_ are reduced compared with pure BiOI (1.85 eV) and TiO_2_ (3.0 eV). A smaller band-gap of bulk BiOI and TiO_2_ will increase the excitation probability of photogenerated electrons, which can stimulate stronger visible light response and enhance the catalytic activity.

As shown in Figure 5b, the valence band maximum (VBM) of the interface is primarily contributed by TiO_2_. The difference VBM between BiOI and TiO_2_ is 0.79 eV. Therefore, the VBO between BiOI and TiO_2_ is 0.79 eV. Here, the CBO is deduced by utilizing the band gaps of BiOI and TiO_2_ from the experiment (3.0 eV for TiO_2_ and 1.85 eV for BiOI). The VBO is calculated by the following formula [37,38,39]:

(3) $$CBO=\Delta E_{gap}^{expt}+VBO$$

$\Delta E_{gap}^{expt}$ represents the difference between the experimental optical bands gap of BiOI and TiO_2_. The CBO calculated by this formula is 1.94 eV.

The VBO can be deduced through the “potential-line-up method” by the following formula [37,38]:

(4) $$VBO=\Delta E_{V}+\Delta V$$

$\Delta E_{V}$ is the difference of the VBM got from the pour structures of BiOI and TiO_2_ under the same pressure. ΔV is obtained through the calculation of the difference of the average of the electrostatic potential through the 1i-BiOI/TiO_2_ heterojunction.

The calculated electrostatic potentials of 1I-BiOI and TiO_2_ are plotted in Figure 5b. The solid red lines represent the average potential of BiOI and TiO_2_ parts. The average electrostatic potentials of BiOI and TiO_2_ are −11.60 eV and −19.96 eV respectively, indicating that ΔV is −8.36 eV. While the VBM of BiOI and TiO_2_ are −3.72 eV and 5.67 eV respectively indicating the calculated $\Delta E_{V}$ is 9.39 eV, so the deduced value of VBO is 1.03 eV. The deduced VBO calculated by the potential-line-up method is about 1.03 eV matching well with the value of the LDOS analysis. As shown in Figure 5b, a built-in potential about 28.3 eV is created around the interface of 1I-BiOI/TiO_2_ junction. Generally, the calculated VBO and CBO of 1I-BiOI/TiO_2_ heterojunction are 0.79 eV and 1.94 eV respectively, which indicates that the BOs between BiOI and TiO_2_ will enhance the separation of photogenerated electron-hole pairs and effectively avoid electron-hole recombination. Therefore, electrons could be transferred from the TiO_2_ to BiOI section and the holes will be transferred in the opposite direction. In addition, the huge difference of potential through the interface capacity to stimulate separation of electron-hole pairs to the farther ends and finally photocatalytic efficiency can be improved.

A substantial charge transfers through the interfaces of 1I-BiOI/TiO_2_ heterojunction. For making a thorough investigation into the electronic distribution and transfer of the 1I-BiOI/TiO_2_ heterojunction, the three-dimensional charge density difference (CDD) is used to analyze the properties of 1I-BiOI/TiO_2_ heterojunction. CDD can describe the “electronic map” vividly and intuitively. This practical method can be deduced by the following formula:

(5) $$\Delta \rho =\rho_{BiOI/TiO_{2}}-\rho_{TiO_{2}}-\rho_{BiOI}$$

Δρ, $\rho_{TiO_{2}}$ and $\rho_{BiOI}$ represent the electronic density of the 1I-BiOI/TiO_2_ heterojunction, the free-standing isolated TiO_2_ and the 1I-BiOI surface in the same configuration, respectively.

**Figure 6:**
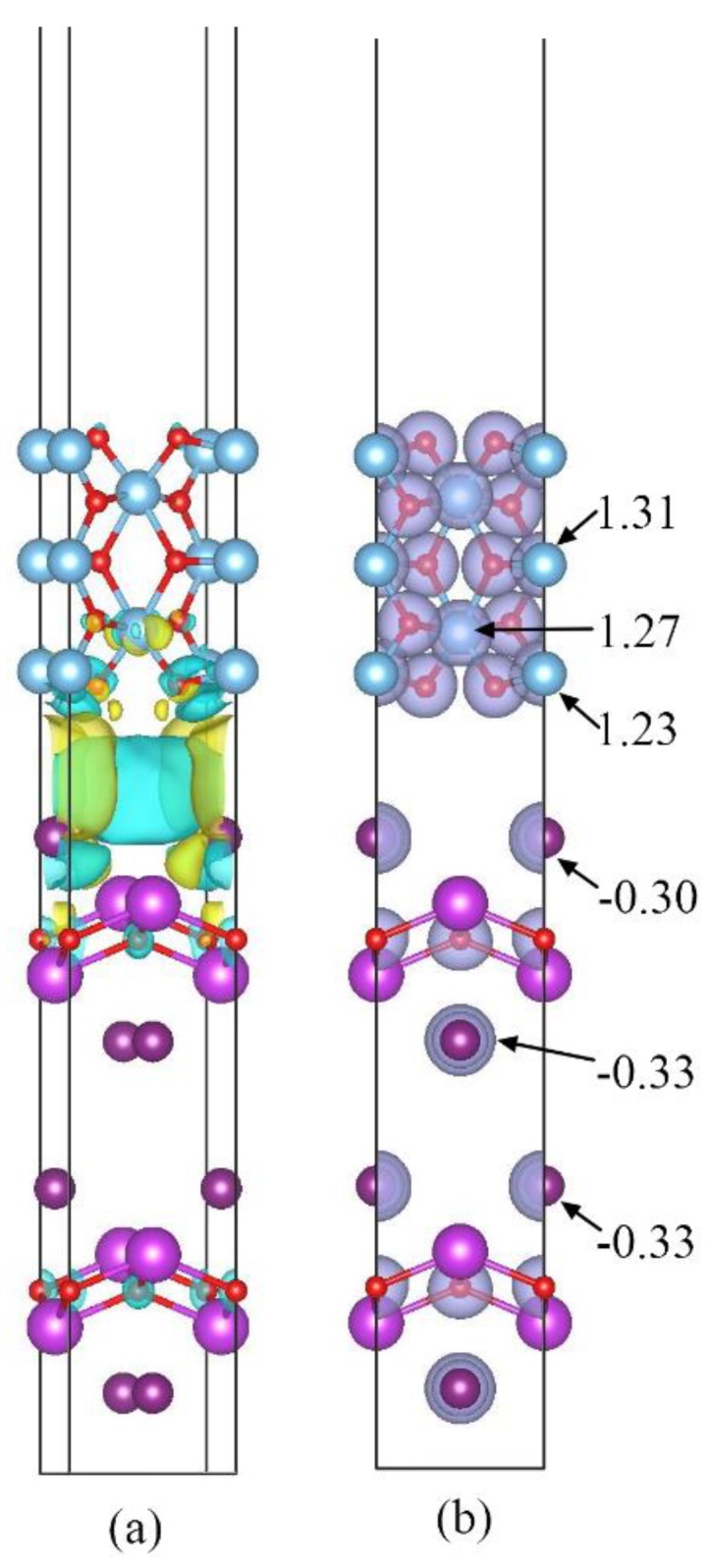
(**a**) 3D charge density difference for the 1I-BiOI/TiO_2_ heterojunction with an isovalue of 0.01 e/Å^3^. Yellow and cyan isosurfaces represent charge accumulation and depletion in the space respectively. (**b**) Mulliken population charge distribution maps of 1I-BiOI/TiO_2_ heterojunction.

From Figure 6a, we found that there is a dramatic change of the electronic density around the interface owing to the combination of TiO_2_ and BiOI. Significant electronic accumulation is observed in the space above I (L-3) atoms in 1I-BiOI part, whereas a little charge depletion is found both on the bottom of Ti (L-2) atoms in the lowest side of the TiO_2_ part and the middle of the interface region of the 1I-BiOI/TiO_2_ heterojunction. The above analysis indicates that the electron transport violently occurs around the interface owing to the combination of BiOI and TiO_2_.

The Mulliken population can present the quantitative calculation of the electronic distribution for the 1I-BiOI/TiO_2_ heterojunction. Here, the Mulliken charge of two representative atoms (I and Ti atoms) is demonstrated in Figure 6b. As shown in Figure 6, the I (L-3) atom obtains a Mulliken charge of −0.30, which is greater than I atoms in other layers of 1I-BiOI part, clearly demonstrating that the electron of I atoms (L-3) increases by a very small amount in the 1I-BiOI/TiO_2_ heterojunction. The charge variation demonstrates that the I (L-3) atom in the top layer of the BiOI part would lose less electrons than I atoms inside the 1I-BiOI part. While the Mulliken charge of Ti (L-2) is 1.23, which is smaller than Ti atoms in other layers, indicating that the electron redistribution appears around the interface. I (L-3) atoms lose some electrons to Ti (L-4) atoms, indicating that the Mulliken charge analysis is consistent with the CDD analysis. Meanwhile, the built-in potential is created around the interface, and the built-in potential drive electronic transfer from the TiO_2_ part to 1I-BiOI part, which is consistent with the band offset analysis in Figure 5a.

#### 3.2.3. Optical Properties and Photocatalytic Mechanism

The dielectric function $\varepsilon (\omega )=\varepsilon_{1}(\omega )+i\varepsilon_{2}(\omega )$ provides a close connection between photons and electrons, and it reflects the linear response of materials to electromagnetic radiation [40]. Absorption spectra can be calculated according to the following formula [41]:

(6) $$\alpha (\omega )=\frac{4k\pi }{\lambda_{0}}=\frac{\omega }{nc}\varepsilon_{2}\left(\omega \right)$$

**Figure 7:**
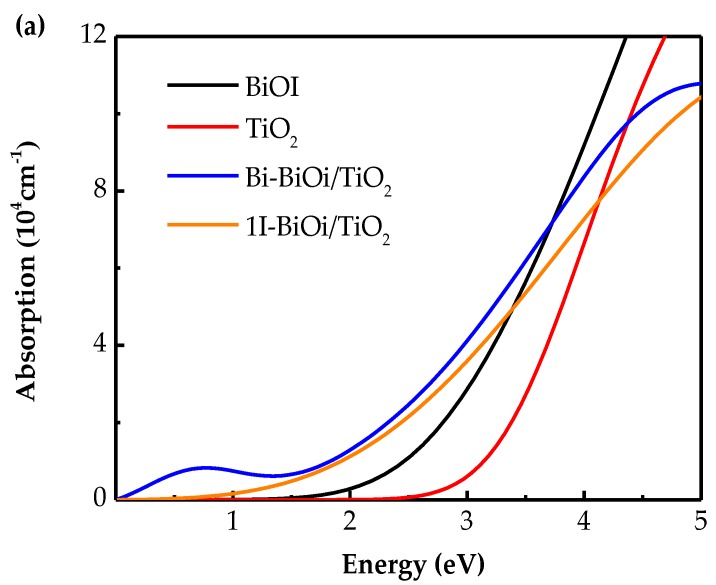

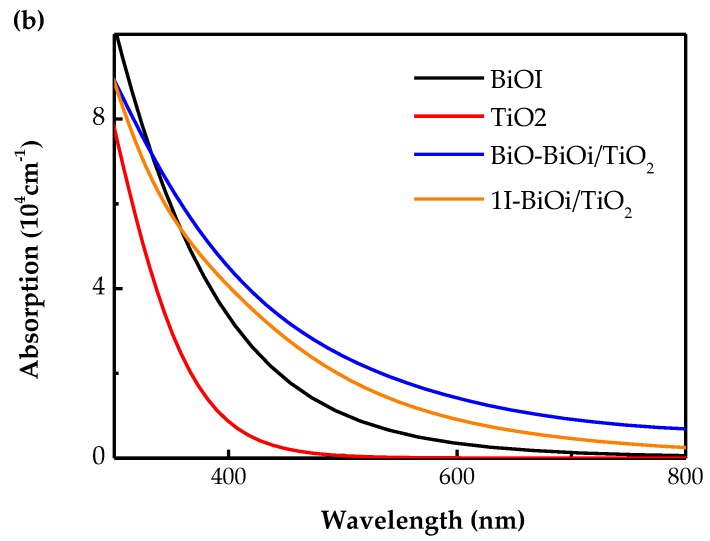
The absorption spectra of pure BiOI, TiO_2_, and BiOI/TiO_2_ heterojunction as the function of (**a**) energy and (**b**) wavelength using the DFT method.

The calculated optical absorption spectra of pure BiOI, TiO_2_ and BiOI/TiO_2_ heterojunctions systems as the function of energy and wavelength are displayed in Figure 7. It can obtain that at the low-energy region from 0 to 1 eV, as shown in in Figure 7a, the optical absorption spectra of BiO–BiOI/TiO_2_ heterojunction has a small characteristic peak at 0.69 eV. The optical absorption amount of BiOI/TiO_2_ heterojunction is much higher than that of pure BiOI and TiO_2_ in the low-energy region. In addition, composing BiOI and TiO_2_ into a heterojunction makes the absorption edges redshift, and the BiO–BiOI/TiO_2_ heterojunction has a larger amount of redshift than 1I–BiOI/TiO_2_ heterojunction. The above analysis makes it clear that the BiOI/TiO_2_ heterojunction can dramatically enhance the performance of absorbing visible light, and finally improve the photocatalytic performance.

**Figure 8:**
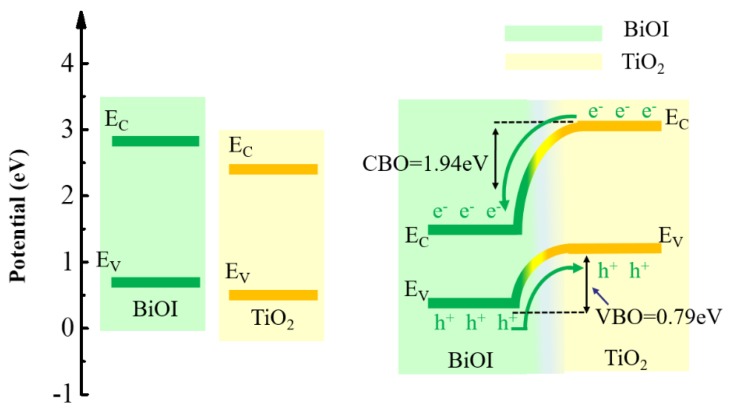
Schematic diagram of the 1I–BiOI/TiO_2_ heterojunction and its energy band distribution.

From the above analysis of the electronic structures and optical properties of the 1I–BiOI/TiO_2_ heterojunction, a schematic diagram of band structure for pre and post binding 1I–BiOI/TiO_2_ heterojunction is shown in Figure 8. We can obtain the conclusions as follows: BiOI with narrow band-gap (1.85 eV) can be tendentiously excited by most of visible light and promote the generation of photoelectrons and holes. However, the generated photo-induced electron-hole pairs of BiOI may recombine immediately due to the narrow band-gap and small sizes, which weakening its photocatalytic properties to a great extent. Because of the large band-gap of TiO_2_ (3.0 eV), TiO_2_ can only use a few parts of visible-light (energy >2.95 eV, wavelength <420 nm). As shown in the schematic diagram of Figure 8, when BiOI and TiO_2_ are contacted with each other constituting a heterojunction, BiOI will play a “photoelectric conversion driver” to absorb visible light. A large built-in potential about 28.3 eV, shown in Figure 5b, and it is created around the interface of the 1I-BiOI/TiO_2_ heterojunction. Therefore, electrons could be pumped from the TiO_2_ to the BiOI section and the holes will transfer in the opposite direction. Meanwhile, the built-in potential capacity will promote photogenerated electron-hole pairs separation on the contrary direction.

As a result, under the light condition (energy >1.85 eV), photogenic positive holes are stimulated in the VB of p-BiOI part priority. The reformed VB edge of BiOI is more inactive than that of TiO_2_ about 0.79 eV. Hence photo-induced holes on the VB of BiOI part would easily transfer to the VB of TiO_2_ part while leaving the photo-induced electrons on the CB of BiOI as shown in Figure 8. Electrons will be driven by transferring from CB of TiO_2_ to BiOI part driven by built-in potential through the surface at the same time. The formed built-in potential at the interface of 1I-BiOI/TiO_2_ heterojunction can significantly accelerate the transfer of photogenerated carriers and prevent its recombination dramatically. The existence of those well-separated photo-electrons and holes would better degrade molecules of organic pollutants. On the whole, the BiOI/TiO_2_ heterojunction possesses excellent photocatalytic properties under the degradation of organic pollutants condition under illumination.

## 4. Conclusions

The characteristics and properties of the 1I–BiOI/TiO_2_ heterojunction and the BiO–BiOI/TiO_2_ heterojunction are studied via the first-principles calculation based on the DFT theory. The structure of the BiOI/TiO_2_ changes slightly, and the interaction on the interface is van der Waals rather than covalent. The band structure of the BiOI/TiO_2_ heterojunction is modified a lot. The band-gap of the 1I–BiOI/TiO_2_ heterojunction reduces to 0.28 eV and the BiO–BiOI/TiO_2_ heterojunction changes into an n-type semiconductor. The calculated VBO of the 1I–BiOI/TiO_2_ heterojunction is 0.79 eV, and the CBO of the 1I–BiOI/TiO_2_ heterojunction is 1.94 eV respectively. The generated electron-hole pairs will be preferentially produced in the BiOI side under light, and then the holes are easily transferred through the interface from the VBM of the BiOI part to the VBM of the TiO_2_ part while leaving the electrons in the CBM of BiOI part. Meanwhile, the built-in potential can effectively speed up the separation of photo-generated electron-hole pairs and effectively avoid electron-hole recombination. Perfectly separated electron-hole pairs across the interface of the BiOI/TiO_2_ heterojunction will greatly improve its photocatalytic performance. The conclusions in this paper can provide a theoretical understanding of the BiOI/TiO_2_ heterojunction and the further utilization of visible-light response materials, such as BiOI-based semiconductor photocatalysts.

## Figures and Tables

**Table 1:** The calculated bond distance (Å) and bond population (e) for the atoms at the interface of the BiOI/TiO_2_ heterojunction.

System	Bond	Bond Distance (Å)		Bond Population (e)
		Before Relaxation	After Relaxation	
1I-BiOI/TiO_2_	Ti (L-2)-I (L-3)	4.06	4.80	0
	O (L-2)-I (L-3)	3.48	4.23	0
BiO-BiOI/TiO_2_	Ti (L-2)-O (L-4)	3.57	3.34	0
	O (L-2)-Bi (L-4)	2.82	2.69	0.08
